# Editorial: Intelligent control and applications for robotics, volume II

**DOI:** 10.3389/fnbot.2023.1282982

**Published:** 2023-10-31

**Authors:** Yimin Zhou

**Affiliations:** Shenzhen Institute of Advanced Technology, Chinese Academy of Sciences, Shenzhen, China

**Keywords:** robotics, perception, decision-making, autonomous control, applications

## 1. Introduction

Robotic technologies have undergone decades of the development and has now entered a highly intelligent stage, which is widely applied in various fields, including production and manufacturing, medical care, education, service Industries (Liu et al., 2020; Omisore et al., 2020). At present, the global robot market has exceeded $100 billion and is growing at a rate of over 17% annually. Among them, the Asia Pacific market is in an absolute leading position, with an estimated expenditure of 133 billion US dollars in 2020, accounting for 71% of the global market.

According to their functions and application fields, the global robot market can be divided into three categories: industrial robots, service robots, and special robots (Yang et al., 2021; Keroglou et al., 2023). Currently, the robotic technologies are being developed with the deep learning and artificial intelligence, where the deep learning technology can enable robots to obtain more accurate artificial intelligence so as to simulate human behaviors.

## 2. Analysis of the Research Topic

“Perception-Decision-Action” is the fundamental framework of the robots. In the perception phase, robots can perceive the environmental information via various sensors such as cameras, LiDAR, and inertial measurement units (IMUs). High-quality perception is crucial for the safe & efficient operation of the robots. The microelectromechanical system (MEMS) IMUs are widely used for the self-localization in the autonomous robots due to their small size and low power consumption. However, they are susceptible to random noise and bias errors, leading to lower measurement accuracy. Liu et al. conducted research on a low-cost MEMS IMU denoising method based on the deep learning. They proposed a hybrid denoising network which can combine the Convolutional Neural Networks (CNN) and Long Short-Term Memory (LSTM) to eliminate the random noise in the raw data and calibrate IMU errors.

With the rapid advancement of the artificial intelligence technologies, vision has become one of the primary modalities for the robot perception (Yang et al., 2020). Robots can use image recognition techniques to extract positional and semantic information from the ambient environment for further decision-making. In practical application scenarios, complex and ambiguous background environments could lead to missed and false detections of small targets. Pei et al. improved the perceptual capability of YOLOv5 for small targets by enhancing input image resolution and retaining more feature information. In addition to the target identification and localization, in some scenarios, robots require to extract more complex semantic information from the visual data. Yang et al. utilized the human joint data, including joint positions, bone vectors, joint motion and bone motion data, to predict the human actions via a multi-scale attention spatiotemporal graph convolutional network. Just as humans can observe and identify objects from different perspectives, robots' active object recognition involves identifying targets through images captured from different viewpoints. Sun et al. developed a sampling strategy and training method for the viewpoint management during the robot perspective transformation process, which can help to determine the optimal planning for the active object recognition.

Planning and decision-making are crucial for the autonomy of the robot systems. The Monte Carlo Tree Search algorithm (MCTS) is a probabilistic search algorithm widely used in the decision-making and path planning problems. MCTS, due to its extensive random searches, is inherently inefficient when addressing individual problems. Li W. et al. introduced a self-learning MCTS (SL-MCTS) by combining MCTS with a dual-branch neural network. Compared with the traditional MCTS, the SL-MCTS is capable of finding better solutions with fewer iterations, significantly enhancing the search efficiency and quality of the path planning tasks. Zhang et al. applied the MCTS in the autonomous decision-making tasks in the aerial combat and incorporated deep reinforcement learning (DRL) to guide the MCTS searching for maneuvers in continuous action spaces, without relying on human knowledge to assist agents in the decision-making.

While DRL demonstrates outstanding performance in the planning and decision-making tasks due to its powerful self-learning capabilities, it might lead to a waste of computational resources when pursuing the maximization of long-term returns in atypical Markov decision processes. Additionally, errors in value function estimation can result in suboptimal policies. Pan et al. addressed these limitations in the atypical MDPs via the average reward method to form an unbiased, low-variance target *Q*-value with a simplified network architecture. Their approach showed significant advantages in terms of learning efficiency, effective control and computational resource utilization compared to the current methods. Meanwhile, Li S. et al. discussed the estimation bias issue, suggesting that a trade-off between the underestimation and overestimation can enhance DRL sample efficiency. They also introduced an Actor-Critic framework, which can learn values and policies within the same network and balances between the underestimation and overestimation.

In the case of multi-agent systems, besides individual autonomous decision-making, the coordinated actions among agents can improve the efficiency of the entire system. Hu et al. explored the problem of minimizing transportation costs in an automated guided vehicle cluster. They employed a hierarchical planning approach to decompose the integrated problem into an upper-level task allocation problem and a lower-level path planning problem. Hence the sum of the request cost and conflict delay cost of the entire system can be minimized via a hybrid discrete state transition algorithm based on the elite solution sets and a taboo list method.

In the execution phase, the robots heavily rely on the control algorithms to perform specific actions (Zheng et al., 2021). Tasks that involve extreme environmental conditions, high workloads and complex operational procedures demand the robots to have a particularly high-level of control precision. Zhao et al. have developed a variable damping controller for the end-effector of a space station robotic arm. With the reinforcement learning to control the variable damping of the robot limb, the resistance of the arm to the disturbance can be greatly enhanced.

## 3. Discussion and conclusion

With the continuous development of mobile robots, technologies such as multi-sensor fusion, control systems, and intelligent software are constantly being upgraded to meet the demands of more application scenarios. According to the statistics analysis, the mobile robot market will be expected to exceed $46 billion by 2025. The Mobile robots have been applied in various fields such as manufacturing, healthcare and military, with enormous potential and unlimited future development space, where the most cutting-edge technologies in the latest robotics field includes: flexible robot technology, liquid metal control technology, electromyography control technology, autonomous driving technology, virtual reality (VR) robot technology, photogenetic technology, brain computer interface (BCI) technology, machine learning (ML) technology, natural language processing (NLP) technology and blockchain technology. These technologies allows a wider range of application scenarios for the development of robots.

Overall, these technologies cover multiple aspects of the robotics field, from robot perception, decision-making to execution, from autonomous learning to interaction with humans, from single perception mode to multimodal perception, from hardware to software, from single decision-making to multi-task collaboration etc. These technologies have all driven the development of the robotics field. Some technologies have been fully developed, such as robot vision technology and robot grasping technology, while others are still rapidly developing, such as robot voice technology and robot navigation technology. Regardless of the technologies, they provide more potentials for the application of the robots and are constantly changing our way of life and work. At the same time, however, robots will also bring new challenges and issues, such as robot ethics, robot laws, robot safety, etc. These issues require us to jointly explore and solve to ensure the health and sustainable development of the robots.

## Author contributions

YZ: Writing—original draft, Writing—review & editing.
